# Editorial: Assessing the Pharmacological Effects and Therapeutic Potential of Traditional Chinese Medicine in Neurological Disease Models: An Update

**DOI:** 10.3389/fphar.2022.909153

**Published:** 2022-04-27

**Authors:** Zhiqiang Deng, Yuxuan Kan, Min Li, Juxian Song, Jia-Hong Lu

**Affiliations:** ^1^ School of Chinese Medicine, Mr. And Mrs. Ko Chi Ming Centre for Parkinson’s Disease Research, Hong Kong Baptist University, Kowloon, Hong Kong SAR, China; ^2^ Medical College of Acupuncture-Moxibustion and Rehabilitation, Guangzhou University of Chinese Medicine, Guangzhou, China; ^3^ State Key Laboratory of Quality Research in Chinese Medicine, Institute of Chinese Medical Sciences, University of Macau, Taipa, China

**Keywords:** stroke, epilepsy, multiple sclerosis, dementia, neurodegenerative diseases

Neurological disorders affect the central and peripheral nervous systems by impairing the function of brain, spinal cord and peripheral nerves. As a wide spectrum of disorders, neurological diseases include epilepsy, stroke, multiple sclerosis, and neurodegenerative diseases, such as Parkinson’s disease (PD), Alzheimer disease (AD) and other dementias. Currently, these disorders have affected hundreds of millions of people worldwide especially in low- and middle-income countries. Traditional Chinese Medicine (TCM) is an ancient yet still widely used medicinal system in East Asia. TCM is an important source in the development of modern drugs. In the past decades, increasing number of studies have investigated the role of TCM-prepartions including medicinal plant extracts or single compounds in neurological disorder models. This Research Topic was launched to summarize the current advances in the application of TCM-originated materials in neurological disorder models and to make a contribution in development of new drugs for the treatment of neurological diseases. The Research Topic has gathered 17 articles consisting of 13 Original Research articles and 4 Reviews articles which have investigated and/or discussed the protective roles of TCM-originated materials in multiple neurological disorders including stroke, depression, multiple sclerosis, epilepsy, and neurodegenerative diseases including PD and AD.

Among the 13 Original Research articles, three have determined the mechanism of TCM effects on stroke. *Luo et al.* investigated the neuroprotective mechanisms of Danggui-Shaoyao-San (DSS), a famous TCM formula including *Angelica sinensis (Oliv.) Diels (Umbelliferae), Paeonia. lactiflora Pall. (Paeoniaceae), Conioselinum anthriscoides “Chuanxiong” (syn. Ligusticum chuanxiong Hort.) (Umbelliferae), Wolfiporia . extensa (Peck) Ginns* (*syn. Poria cocos (Schwein.) (Polyporaceae), Atractylodes macrocephala Koidz. (Asteraceae),. and Alisma plantago-aquatica subsp. orientale (Sam.) Sam.* (*syn. Alisma orientalis (Sam.) Juzep. (Alismataceae)*, for the treatment of ischemic stroke (Luo et al.). By administrating ethanol extract of DSS into rats which were subjected to 2 h of MCAO (middle cerebral artery occlusion) plus 22 h reperfusion, they found that ethanol extract of DSS can reduce infarct sizes and improve neurological deficit scores in these rats. Mechanistically, they found that ethanol extract of DSS can inhibit the expression of p67^phox^, a subunit of NADPH. Meanwhile, DSS up-regulates SIRT1 expression in the cortex and striatum of MCAO ischemic brains which can be ablated by SIRT1 inhibitor EX527. Together, they demonstrated that DSS protects against cerebral ischemic-reperfusion injury through SIRT1-dependent manner. By using the similar MCAO model of stroke, Wang et al. found Taohong Siwu decoction (THSWD) containing Tao Ren (*Prunus persica* (*L.*) Batsch), Hong Hua (*Carthamus tinctorius* L.), Dang Gui (*Angelica sinensis* (Oliv.), Shu Di Huang (*Rehmannia glutinosa* (Gaertn.)), Chuan Xiong (*Ligusticum chuanxiong* Hort), and Bai Shao (*Paeonia lactiflora* Pall.) can improve the behavioral function and pathological damage of brain in MCAO rats (Wang et al.). They further demonstrated that THSWD could reduce the activity of NLRP3 inflammatory corpuscle by down-regulating the expression of inflammatory factors and inhibit pyroptosis pathway in MCAO rats. As a classical TCM, *l*-borneol has been used to treat stroke in China for thousands of years. Ma et al. investigated a novel mechanism of *l*-borneol’s effect on stroke (Ma et al.). They found that *l*-borneol improves the neurological deficits and pathological damage of cerebral ischemia in MCAO rats through promoting angiogenesis and neurogenesis. Mechanistically, they found that *l*-borneol can increase the number of CD34 positive cells and decrease the levels of ACE and Tie2 to promote angiogenesis, and can significantly enhance the expression of VEGF and BDNF while inhibit the expression of TGF-β1 and MMP9 to promote neurogenesis. They further verified the mechanism by using molecular docking assay showing a very high binding rate between *l*-borneol and the targets above.

Five of 13 Original Research articles have determined the mechanism of TCM effects on depression. *Li et al.* investigated the curative effect and mechanism of volatile oil from *Aquilaria sinensis* (*Lour.*) *Gilg* and *Aucklandia costus Falc.* (CMVO) in the treatment of depression (Li et al.). In chronic unpredictable mild stress (CUMS) rats, a depressive rat model, they found CMVO displayed an antidepressant effect based on the multiple animal behavior tests. They showed that inhalational administration of CMVO to CUMS rats decreased the level of adrenocorticotropic hormone in serum through down-regulating the expression of corticotropin-releasing hormone mRNA in hypothalamus, whereas CMVO restored the level of 5-hydroxytryptamine (5-HT) in the hippocampus through up-regulating the expression of 5-HT_1A_ mRNA. Qu et al. reported on the role of Kai-Xin-San (KXS), a TCM formula composed of Ginseng Radix et Rhizoma, Polygalae, Acori Tatarinowii Rhizoma, and Poria, in the regulation of neuronal inflammation in mouse model of CUMS (Qu et al.). They confirmed the antidepressant effects of KXS in CUMS mice. In mechanism, they demonstrated that KXS inhibited the activation of microglia by reducing the expression of pro-inflammatory cytokines (IL-1β, IL-2 and TNG-α) in hippocampus of CUMS mice. Zhu et al. evaluated the antidepressant effect of Elaphuri Davidiani Cornu (EDC) in depression-like mouse model (Zhu et al.). They found that aqueous extracts of EDC can significantly improve depression-like behavior and enhance the expression of nerve growth factors and brain-derived neurotrophic factors in prefrontal cortex and hippocampus. In the primary cultures of astrocyte derived from these depression-like mice, they identified that the EDC aqueous extracts exerted the antidepressant effect through cAMP- and ERK-dependent pathways. By using an olfactory bulbectomized (OB) rat model of depression, Ji et al. investigated the antidepressant-like effects of Xiaoyao pills (XYW) composed of paeoniflorin, liquiritin, saikosaponin B2, and atractylenolide Ⅱ. and the underlying mechanism (Ji et al.). According to the results of multiple behavior tests in the OB rats treated with XYW, they found XYW significantly alleviated the depression-like behaviors. They further demonstrated that XYW played these protective roles by inhibiting oxidative stress and enhancing the activation of PIK3CA-AKT1-NF2EL2/BDNF signaling pathways. Zhao et al. determined the protective function of Jiedu Tongluo granules (JDTLG, which was composed of *Panax ginseng C. A. Mey*. (Ren Shen), *Scutellaria baicalensis Georgi* (Huang Qin), *Ginkgo biloba L*. (Yin Xing Ye), *Hypericum perforatum L* (GuanYe Lian Qiao), *Gardenia jasminoides J*. *Ellis* (Zhi Zi), *Gastrodia elata Blume* (Tian Ma), *Conioselinum anthriscoides* “Chuanxiong” (Chuan Xiong.) in post-stroke depression (PSD) rat model established by catotid artery embolization combined with chronic sleep deprivation (Zhao et al.). They found that the neurological deficit and depression symptoms of PSD rats can be significantly improved by oral treatment of JDTLG. By performing proteomic analysis of the brain tissue of PSD rats, they identified several processes including N-methyl-D-aspartate receptor (NMDAR) and brain-derived neurotrophic factor (BDNF) signal pathway involved in the regulation of JDTLG on PSD rats.

Three of 13 Original Research articles have determined the effects and mechanisms of TCM on neurodegenerative diseases including PD and AD. Chen et al. reported on the neuroprotective effect of corynoxine, an oxindole alkaloid isolated from the Chinese botanical drug Uncaria rhynchophylla (Gouteng in Chinese), on animal models of PD (Chen et al.). They demonstrated that corynoxine can improve motor dysfunction, prevent tyrosine hydroxylase (TH)-positive neuronal loss, and decrease α-synuclein aggregation in rotenone-induced rat and mouse models. These results will provide experimental basis for corynoxine in the treatment of PD. Huang et al. investigated the effects and potential mechanisms of berberine on NLRP3 inflammasome in PD (Huang et al.). By using *in vivo* models (MPTP-induced PD mice), the authors found that berberine enhanced autophagy and mitigated behavioral impairments, neurotoxicity and neuroinflammation. The *in vitro* data confirmed the inhibitory effect of berberine on NLRP3 inflammasome, including the expressions of NLRP3, cleaved caspase 1 (CASP1), and mature interleukin 1 beta (IL1B). In addition, treatment with the autophagy inhibitor 3-Methyladenine (3-MA) blocked the effect of berberine both *in vivo* and *in vitro*. These results supported the neuroprotective effect of berberine on PD, and provided the mechanism by which berberine inhibits NLRP3 inflammasome activation through enhancing autophagy. Iyaswamy et al. reported on the effect of Chinese medicine formula Yuan-Hu Zhi Tong (YZT), composed of *Corydalis yanhusuo* and *Angelica Dahurica*, on AD (Iyaswamy et al.). In P301S tau and 3XTg-AD mice, they found that YZT can reverse motor dysfunction and enhance learning and memory function, respectively. By using Microarray and the Connectivity Map analysis, the authors determined that YZT reduced tau aggregation by regulating ubiquitin proteasomal system. The study suggests YZT can be a potential drug for the treatment of AD.

Among the 13 Original Research articles, Sun et al. attempted to illuminate the mechanism of catalpol in the treatment of multiple sclerosis (Sun et al.). The authors established cuprizone-induced demyelination model and found that catalpol improved the motor functions and promoted myelination in the model. Both *in vivo* and *in vitro* data showed that catalpol promoted the differentiation of oligodendrocyte precursor cells (OPCs), which are critical for the formation of remyelination in multiple sclerosis. Mechanistically, they found that the effect of catapol on remyelination may be related to the inhibition of NOTCH1 pathway. This study provided a mechanistic rationale for catapol in the treatment of multiple sclerosis. Tian et al. reported on the mechanism of Chinese medicine Lvjiaobuxue granule, an immunomodulator, against acute leukopenia (Tian et al.). The results showed that Lvjiaobuxue granule improved the blood routine parameters and organ index (including spleen, thymus and liver) in cyclophosphamide-induced leucopenia model of 4T1 tumor-bearing mice. According to the analysis of metabolomics and network pharmacology, the regulation of branched-chain amino acids (BCAAs) degradation may play a pivotal role in mice with leukopenia after Lvjiaobuxue granule treatment. This study provided data and theoretical support for further research on its mechanism.

Among the 4 Review articles, two of them reviewed the neuroprotective effects of natural products on ischemic stroke. Xie et al. comprehensively summarized the pharmacological effects and the underlying mechanisms of natural products for cerebral ischemic injury in multiple preclinical models, and discussed their potential applications in neuroprotection (Xie et al.). They proposed the potential role of the structures of natural products in their biological activity in neuroprotection, which have not been determined currently. The other one contributed by Li et al. systematically reviewed the neuroprotective effects of borneol and the mechanisms of the actions in ischemic stroke at different stages including acute stage, subacute stage and late stage (Li et al.). By performing mata-analysis on key indicators in the experiments *in vivo*, they concluded that unlike many other drugs, borneol protects against neuronal injury in ischemic stroke via multiple mechanisms at different stages, hastens self-repair of the body by mobilizing endogenous nutritional factors, and enhances the therapeutic effects of other drugs by promoting them to pass through the blood-brain-barrier (BBB). Among the 4 Review articles, one of them is related to the treatment of epilepsy by natural medicine (He et al.). In this review article, He et al. reclassified the ingredients of certain natural medicines and discussed their antiepileptic mechanisms, which would provide benefits to drug development for epilepsy treatment. The last review article is contributed by (Long et al.). In this article, they reviewed the role of PI3K/AKT signal pathway in AD and PD, and summarized the natural products which are displayed preventive and therapeutic effects on these two diseases via PI3K/AKT pathway. The review article would provide guidance and reference for the development of novel drugs for the treatment of AD and PD in this field.

In summary, despite the research quality in these studies are improved by using advanced technologies and powerful tools of molecular biology, several issues remain unsolved currently. For instance, the particular molecular mechanisms by which the TCM-preparations exert protective effects against neurological disorders are still unclear. In the future, research can be benefited from applying the promising approaches including but not limited to using unbiased omics-based analysis to comprehensively decipher the mechanism; using transgenic animal models to significantly enhance the disease relevance; and systemically verifying the drug targets in multiple animal models.

